# Scaling Up and Sustaining Voluntary Medical Male Circumcision: Maintaining HIV Prevention Benefits

**DOI:** 10.9745/GHSP-D-16-00159

**Published:** 2016-07-02

**Authors:** Emmanuel Njeuhmeli, Marelize Gorgens, Elizabeth Gold, Rachel Sanders, Jackson Lija, Alice Christensen, Francis Ndwiga Benson, Elizabeth Mziray, Kim Seifert Ahanda, Deborah Kaliel, Tin Tin Sint, Chewe Luo

**Affiliations:** a United States Agency for International Development, Washington, DC, USA; b The World Bank, Washington, DC, USA; c Johns Hopkins Center for Communication Programs, Baltimore, MD, USA; d Avenir Health, Project SOAR, Washington, DC, USA; e Ministry of Health and Social Welfare, Dar es Salaam, Tanzania; f Jhpiego, AIDSFree Tanzania, Dar es Salaam, Tanzania; g Ministry of Health, Nairobi, Kenya; h United Nations Children’s Fund (UNICEF), New York, NY, USA

## Abstract

To maintain high circumcision prevalence, voluntary medical male circumcision programs in East and Southern Africa need to plan for sustainability and conduct transition assessments early on, rather than waiting until the saturation of priority targets at the end of the program.

## Teaser

To maintain high circumcision prevalence, voluntary medical male circumcision programs in Eastern and Southern Africa need to plan for sustainability and conduct transition assessments early on, rather than waiting until the saturation of priority targets at the end of the program.

## INTRODUCTION

The changes in the AIDS financing landscape and in the architecture of health financing under the post-2015 sustainable development agenda have highlighted the need to ensure that HIV and AIDS programs continue to yield health benefits regardless of funding sources, implementation mechanisms, and governance structure. Further highlighting this need are the changes in the nature of institutions implementing HIV services and the number of people who will need HIV services to end AIDS by 2030. Thus, donors in the international development arena have increasingly mandated that the interventions of their funded programs be sustainable.

Yet sustainability—sometimes called institutionalization^1^—can mean different things in different contexts. According to the U.S. President’s Emergency Plan for AIDS Relief (PEPFAR), “a sustainable response can only be achieved when the epidemic is under control and no longer expanding and the response is constantly adapting to the evolution of the epidemic to maintain control.”^2^ In the context of voluntary medical male circumcision (VMMC), this article defines a sustainable VMMC program as one whose local stakeholders maintain high circumcision prevalence after the initial scale-up—generally by incorporating either early infant male circumcision (EIMC), early adolescent VMMC, or both, into routine newborn and adolescent service delivery systems.^3^ (EIMC is performed during the first 60 days of an infant’s life and early adolescent VMMC between the ages of 10 and 14.)

In a sustainable VMMC program, local stakeholders maintain high circumcision prevalence by incorporating EIMC, early adolescent voluntary medical male circumcision, or both into newborn and adolescent service delivery.

In December 2011, the “Joint Strategic Action Framework to Accelerate the Scale-Up of VMMC,”^4^ launched by the World Health Organization (WHO) and the Joint United Nations Programme on HIV/AIDS (UNAIDS), articulated a 5-year strategy to achieve at least 80% circumcision coverage (i.e., saturation) among males ages 15 to 49 in 14 priority countries in Eastern and Southern Africa with generalized HIV epidemics, high HIV prevalence, and low prevalence of male circumcision. By the end of 2014, the 14 countries had reported nearly 9 million VMMCs, with some countries about halfway toward reaching their targets and others lagging behind.^5^ However, the number of circumcisions and total coverage do not provide the whole picture. In some countries, progress has been uneven, and certain age groups and certain districts are reaching saturation before others.

Based on the initial targets, these regions will need to start planning for sustainability sooner than originally thought. Tanzania, which has prioritized 11 regions for VMMC, is an example of this unevenness. The Iringa and Njombe regions are close to saturation among males ages 15 to 24, while other regions lag behind. As Iringa and Njombe regions achieve their targets for saturation, the time is now to consider planning for sustainability: preparing to transition the regions’ male circumcision activities to local stakeholder design, management, and funding and to include young adolescents and infants as part of the target age range. Transition planning includes VMMC becoming institutionalized into the health care infrastructure of Tanzania. In this article, we discuss critical considerations for VMMC sustainability and highlight sustainability approaches taken by 2 countries—Kenya and Tanzania.

## SUSTAINING HIV/AIDS PROGRAMS: FINANCING AND SERVICE DELIVERY

Core to all definitions of sustainability is the notion that the ongoing program will continue to yield an agreed-upon set of health benefits, and those programs will be planned, managed, and eventually funded by local stakeholders, including government, private sector, civil society, and others. Current thinking and lessons learned from transitions in other programs (such as U.S. Agency for International Development [USAID] graduations in family planning^6^ and the Bill & Melinda Gates Foundation’s transition of the Avahan HIV prevention program in India from donor to government financing^7^) offer the following lessons about sustainability (or continuation):

- Early planning is critical to successful transitions—planning for sustainability and transition needs to be factored in from program inception, not just when financing is shifting or phasing out.
- Sustaining the status quo is not a prerequisite. Transition could also involve moving to more efficient and effective service delivery modalities and better targeting programs to populations and geographical areas that are most important to the HIV response for a particular context. It is a dynamic process and plans should be adjusted as needs shift.^8^
- Clear communication among all related stakeholders is critical for success.
- Technical and managerial support is often needed to build domestic capacity and ensure the institutionalization of support mechanisms.
- A systematic, phased approach to transition planning allows for course corrections and helps ensure that critical elements are considered.
- Post-transitional support is important to ensure quality as well as to assess transition effectiveness.
- Transition planning might result in changes in implementing agencies (moving from service delivery by an NGO to government service delivery, for example) or changes in governance approaches (moving from coordination and budgeting by donors to coordination by government departments).

### Donor Focus on Sustainability of HIV Programs

Building on these lessons, several partners, including the World Bank, PEPFAR, and the Global Fund to Fight AIDS, Tuberculosis and Malaria, have embarked on efforts to support countries in planning for and improving their programs’ sustainability. As PEPFAR enters its third phase, there is a new “Sustainability Action Agenda,” which includes, among other strategies, implementing the “Sustainability Index and Dashboard” (a tool designed to measure the sustainability of national HIV responses across 4 domains with approximately 80 indicators) in PEPFAR country programs; building on health systems strengthening and human resources for health programs; and engaging with multilateral institutions and civil society for greater coordination on sustainability. The World Bank is supporting countries to identify the constraints to and opportunities for developing health financing systems to accelerate and sustain progress toward universal health coverage.^9^ The Global Fund has just released a policy paper on sustainability, transition, and co-financing.^10^

### HIV Sustainability Frameworks and VMMC Sustainability

To ensure the sustainability of HIV/AIDS results, certain factors are important to assess, plan for, and strengthen. Drawing on the 4 domain areas of the PEPFAR Sustainability Index and Dashboard as a way to frame the discussion, we note these areas as critical for VMMC sustainability:

- **Governance, leadership, and accountability:** Coordination, led by government, across all donors and stakeholders is essential for long-term program success and sustainability. New policies may be needed, such as national or subnational strategies and implementation plans, or legal actions may be needed (e.g., formation of new institutions or bodies, or enabling laws) to ensure that practices are formalized and changes in governments or leadership do not mean gains are lost. In some cases, policy changes affect the institutions responsible for managing the HIV/AIDS response or the governance structure more broadly. In addition, ensuring engagement from civil society and the private sector as program planners, implementers, and monitors is key, with the assurance that all stakeholders have access to information in a transparent manner that allows for holding each other accountable for results.
- **National health system and service delivery:** Changes in financing sources may result in the need to change service delivery modalities (e.g., to shift from service delivery by an NGO to public-sector service delivery). Before planning the transition, it is important to understand which services are essential, and for which populations, in which geographical areas, and at what intensity. For example, as targets are reached for VMMC coverage for men 15 to 49 years old, there may be a need to consider targets for other age ranges. Along with direct service delivery, systems support is critical to successful service delivery, for instance, ensuring sufficient numbers of health care providers are trained, commodities and logistics systems are reliable, and laboratory systems are functioning. For long-term success, high-quality and responsive service delivery is also key to meeting the demand of clients and should be assessed.
- **Strategic investment, efficiencies, and sustainable financing:** Shifts in the source or level of financing may have implications for the type and level of services to be delivered and their sustainability. Planning transitions necessarily involves understanding the current and future financial landscape, including the possibility of assigning public resources for specific priority services, and the opportunities for diversified funding through resource mobilization and innovative financing from both public and private sources. The landscape may require advocating inclusion of VMMC in the national health insurance basic benefit package. In the context of reduced financing, and because of the need to focus on more efficient HIV responses, it is also important to look at allocating resources across populations, programs, and geographical areas to achieve the highest impact at the lowest cost. The potential for integrating services should be considered.
- **Strategic information:** Institutionalized, regular data collection is needed to monitor the progress of programming. Data should include specific surveys and surveillance, expenditures, and performance measures. Data should be made available in a timely manner so they can be used to better inform and target programming.

The 4 domain areas of the PEPFAR Sustainability Index are governance, leadership, and accountability; national health system and service delivery; strategic investment, efficiencies, and sustainable financing; and strategic information.

## WHAT DOES VMMC SUSTAINABILITY ENTAIL?

As noted above, VMMC sustainability involves local stakeholders maintaining high circumcision prevalence through EIMC and/or early adolescent VMMC and integration into service delivery systems. Two considerations are critical to ensuring VMMC sustainability:

- VMMC age-group prioritization after adult saturation
- The status of the national VMMC response for sustainability

### VMMC Age-Group Prioritization

Depending on the context and desires of the local government, it may make sense to focus on different populations as there is near-saturation of the 15 to 49 age range during or after scale-up and the program transitions to local stakeholder ownership. In the scale-up phase, the country circumcises males across the entire target age group, requiring a large number of annual circumcisions. After the target coverage is achieved, males are circumcised only as they move into the focus age group to maintain the target coverage level. These focus population groups are:

- **Adolescents:** The country continues to circumcise adolescents ages 10 to 14 to maintain target coverage.
- **Infants:** Local stakeholders start to increase EIMC during or after the larger adult and adolescent scale-up phase. It takes about 12 years for infants circumcised during the scale-up phase to age into the 10 to 14 age group; during that time, the country must keep circumcising adolescents to maintain coverage. But once the infants reach adolescence, it is no longer necessary to continue circumcising adolescents, and the country can focus entirely on EIMC.
- **Adolescents and infants (mixed focus)**: The country focuses on both infants and adolescents, since high coverage among infants is never achieved and both adolescents and infants will need to be circumcised indefinitely to maintain the target coverage across the population.

### The Sustainability Status of the National VMMC Response

To plan for a responsible transition and long-term sustainability, an assessment is suggested that includes 15 elements drawn from the PEPFAR Sustainability Index and Dashboard. The 15 elements are:

- Policy
- Planning and coordination
- Civil society engagement
- Private-sector engagement
- Public access to information
- Quality
- Service delivery
- Human resources for health
- Commodity security
- Laboratory
- Financing
- Efficiencies
- Surveys and surveillance
- Expenditure data
- Performance data

To plan for a responsible transition and long-term sustainability, the 15 elements drawn from the PEPFAR Sustainability Index and Dashboard should be assessed.

After an assessment of these elements, weak elements should be strengthened before a program is transitioned. Financing and tracking domestic expenditure on VMMC programs is important, although hard to monitor because of the way government charts of accounts are developed, and because VMMC delivered in primary care settings would not necessarily be listed as a separate line item in a budget or district hospital expense report.

We discuss below some of the elements that have been considered for VMMC transitions.

#### Human Resources and Commodities

Human resources, commodities, and supplies are as important as financial resources for health care services. A preliminary analysis by the USAID-funded Project SOAR (Supporting Operational AIDS Research) explored the human resources and commodity costs in Tanzania for the focus populations described earlier.^11^

Project SOAR calculated the human resource requirements to achieve service goals based on the number and age of people to be circumcised and on provider time required per person (based on country costing studies). The need for doctors and nurses depends on both client numbers and treatment inputs for care. Because EIMC was seen as requiring only 20 minutes of doctor time,^11^ the EIMC scenario results in a much lower need for doctors, even though the number of individuals to be circumcised is not much lower than in other scenarios. If Tanzania’s 2013 population of 36 trained doctors^12^ remains constant, it will not be possible to achieve the goals of any of the 3 scenarios without more training or task shifting. A more positive picture emerges when nurses and midwives are considered. With 853 trained nurses available,^13^ all 3 population scenarios are achievable; there is no human resource constraint when this level of staff is involved (although the EIMC scenario would require an emphasis on midwives).

When the focus is on EIMC, analysis by SOAR reveals that drug and supply costs are around US$6.9 million in year 1, rising to about US$7.6 million by year 6. When the strategic focus is on adolescents, the costs are a bit less, primarily due to the lower service numbers, beginning around US$4.6 million for year 1 and rising to US$5.8 million in year 6. The mixed-focus strategy is the most expensive for drugs and supplies. Its cost begins around US$8 million for year 1 and increases to almost US$10 million by year 6.

From this preliminary analysis, we reached the following conclusions with regard to human resources for health and commodity costs:

- Sustaining VMMC programs can challenge countries, but in the cases considered in this analysis, most resources suffice if a task-shifting policy is implemented.
- Scale-up and sustainability of VMMC could burden the health system if it demands specialists or a large number of providers.
- Additional analysis is needed, particularly around other health system issues such as the supply chain, but also across all 15 elements of the PEPFAR Sustainability Index.

Sustaining VMMC programs can challenge countries, but in the cases considered in this analysis, most resources suffice if a task-shifting policy is implemented.

#### Demand Considerations for VMMC Sustainability

As countries transition from the current catch-up model, with demand-creation efforts focusing on males ages 15 to 29, to one of the three population-focused scenarios described earlier, new communication strategies for demand creation will be necessary. Regardless of the sustainability path to meet targets efficiently, a country’s program must be supported by a communication strategy to ensure that demand matches supply. An overarching communication component would address common needs of early adolescent VMMC and EIMC to ensure coordination, prevent conflicts between the two, and address the potential addition of resources and channels that may not have been part of the catch-up approach.

For example, under the infant-focus strategy, EIMC education needs to be linked to a country’s maternal and child health and antenatal programs. Capacity strengthening of staff charged with client education and counseling will likely be required. Formative research will be needed to shape context-specific strategies and approaches that address the barriers to EIMC and the motivating factors inherent in the sociocultural environment. Although considerable knowledge and positive norms exist around VMMC, and although EIMC builds on VMMC platforms, EIMC is new to many populations in East and Southern Africa, and knowledge and experience around EIMC are not as extensive as for adult VMMC and even for early adolescent VMMC.

#### What Do We Know About Demand for EIMC?

Since we have more experience in generating demand for VMMC among the early adolescent population and limited experience with EIMC, we conducted a rapid literature scan on demand for EIMC. Using varied methodologies, we found 11 studies conducted between 2010 and 2015 in 6 countries (Botswana, South Africa, Swaziland, Tanzania, Zambia, and Zimbabwe). Three studies were pilots looking at factors influencing EIMC service uptake.^14^^-^^16^ Six studies, all theoretical, looked at EIMC acceptability in the absence of current EIMC services.^17^^-^^22^ One study, conducted in 2014, was a systematic review and thematic synthesis looking at factors associated with parental decisions not to use EIMC services.^23^ Although this literature review was not comprehensive, common themes emerged.

**Key barriers:** One recurrent theme was the lack of accurate information and poor knowledge about the EIMC procedure and its advantages. Men projected their own experience and pain with VMMC and traditional circumcision and failed to differentiate between VMMC and EIMC. Safety was a common concern, particularly the fear that EIMC would injure or irreparably damage the newborn’s penis, perceived as too fragile for the procedure. Fear of infant death and excessive bleeding emerged as more immediate concerns, along with concerns about future effects of EIMC, including decreased penile sensitivity, more sexual risk-taking by circumcised men, and ostracism or rejection by peers. Studies also looked at parental preferences for circumcision timing, which often conflicted with the recommended window (before 2 months of age). In some cases, these parental preferences were linked to the timing of traditional rites of passage, while in other cases preferences were associated with beliefs around the fragility of the infant penis. Sociocultural beliefs and myths are also obstacles to demand for EIMC, including fears that the baby’s discarded foreskin would be used for satanic or other malicious purposes. Another critical hurdle identified in most studies was lack of support by fathers, who are key decision makers on circumcision. Although mothers and grandparents also play a role, fathers have the final say.

One recurrent theme of our literature scan of demand for EIMC was the lack of accurate information and poor knowledge about the EIMC procedure and its advantages.

**Motivating factors:** Factors motivating EIMC uptake were: availability of EIMC services at no charge; recognized protection from HIV and sexually transmitted infections; improved hygiene; availability of highly trained personnel performing the procedure; word-of-mouth recommendation from satisfied parents; and the belief that infants heal faster and have less awareness of the procedure than older boys.

**Key recommendations:** To generate demand for EIMC, key recommendations emerging from the literature scan included:

- Strengthen knowledge of EIMC in the community as well as among parents.
- Include fathers directly among the key audiences for health communication.
- Target multiple generations, as grandparents are influencers.
- Time education early enough to allow for family discussion and planning.
- Use satisfied parents strategically in communication efforts.
- Develop materials for service providers’ use (with clear information about the procedure).
- Address the sociocultural factors in each local context (e.g., parental age preferences for circumcision).

## SUSTAINABILITY APPROACHES IN KENYA AND TANZANIA

Kenya and Tanzania have made great progress in meeting initial male circumcision targets and are in the process of transitioning to sustainability. As both countries have made gains economically, they are also under pressure to increase domestic resources for HIV overall.

### Tanzania

The government of Tanzania introduced VMMC for HIV prevention in 11 priority regions in 2010 and set a target of 2.8 million circumcisions by 2016.^24^ Through the end of 2015, more than 1.3 million VMMCs were conducted.^25^ Two traditionally non-circumcising regions, Iringa and Njombe, made substantial progress toward their regional targets and reached complete VMMC saturation among adolescents (Table). In 2010, Njombe was part of the Iringa region and the regional target for Iringa/Njombe was 265,000 for 2010–2015. (In 2012, Njombe split off from Iringa region.) By the end of 2015, 173,362 VMMCs had been performed in Iringa and 130,118 in Njombe, totaling 303,480.^27^ Because they reached saturation in these 2 regions, Iringa and Njombe are now ready to pilot a sustainability approach that can help guide transition strategies for the other regions in Tanzania. The 3-pronged approach that Iringa and Njombe are piloting includes: (1) transitioning service delivery from donor-funded partners to the government; (2) targeting clients entering adolescence; and (3) scaling up EIMC services.

**TABLE:** Progress Toward Target VMMC Coverage (%) in 11 Priority Regions of Tanzania by Age Group, 2010–2014

Region	10–14 Years		15–19 Years		20–24 Years		25–29 Years		30–34 Years		35–49 Years		15–49 Years	
	2010	2014	2010	2014	2010	2014	2010	2014	2010	2014	2010	2014	2010	2014
Geita	10	35	16	38	22	31	22	27	23	26	23	29	**21**	**31**
Iringa^a^	15	100	32	99	35	72	24	55	28	46	26	40	**29**	**63**
Kagera	19	40	34	55	40	55	36	48	44	48	39	48	**38**	**51**
Katavi	12	31	27	47	30	44	23	37	18	33	21	29	**24**	**38**
Mbeya	15	60	33	71	36	55	25	44	29	39	26	35	**30**	**49**
Mwanza	28	45	42	59	59	68	58	65	62	66	62	75	**56**	**67**
Njombe^a^	15	100	32	100	35	97	24	68	28	53	26	43	**29**	**80**
Rukwa	12	40	27	52	30	43	23	36	18	31	21	28	**24**	**38**
Shinyanga	10	49	18	67	22	66	20	49	24	38	21	31	**21**	**50**
Simiyu	10	47	18	58	22	55	20	41	24	34	21	30	**21**	**44**
Tabora	19	49	33	63	40	59	36	51	43	51	39	49	**38**	**55**
**All 11 priority regions**	**20**	**55**	**40**	**75**	**49**	**70**	**41**	**60**	**46**	**55**	**43**	**54**	**44**	**63**

Abbreviation: VMMC, voluntary medical male circumcision.

a Iringa and Njombe are traditionally non-circumcising regions that have made substantial progress toward their regional targets, including reaching complete VMMC saturation among adolescents.

Sources: Tanzania HIV/AIDS and Malaria Indicator Survey 2007-08^26^ and DMPPT (Decision Makers’ Program Planning Tool) 2.1 modeling by Project SOAR (Supporting Operational AIDS Research).

Transitioning service delivery from donor-funded partners to government can be done in several ways. In one option, as donors transition programming, the government picks up funding and provides services directly through their facilities and staff. Alternatively, as the government picks up funding, it can guarantee long-term sustainability by funding NGOs and contracting out services to private providers. Or, in a combination of the 2 mechanisms, NGOs and private providers are integrated into the public-sector financing and delivery mechanism. For Iringa and Njombe, transitioning service delivery from donor-funded implementing partners to service providers funded by the government involves such activities as increasing the number of static service delivery sites, supporting district-led outreach and mobile services, shifting commodities from disposable to reusable, and integrating VMMC commodity requirements into the current national supply chain. These components and others require an assessment to evaluate status and plans to increase capacity or strengthen the system before full transition.

The second prong involves clients entering adolescence (i.e., beginning at age 10). This approach requires age-appropriate services and counseling, on-the-job training for all VMMC providers on making services adolescent friendly, and linking with schools and youth activities to create demand among adolescents.

The third prong of Iringa’s and Njombe’s sustainability approach focuses on scaling up EIMC services. Since 2013, more than 3,800 EIMCs have been conducted at 8 pilot sites in the Iringa region.^24^ The pilot uses an integrated model, in which EIMC services are offered as part of child health services.

### Kenya

Kenya rolled out VMMC in late 2008 when it published its first VMMC strategy (2008–2013).^28^ The goal was to increase national VMMC coverage from 85% to 94% by circumcising 860,000 men. Circumcision rates vary by province, however, and in Nyanza, the region with the highest HIV prevalence, male circumcision coverage is lower than the national average. The baseline male circumcision prevalence in 2009 for males ages 15 to 49 in Nyanza region was 44.8%, compared with 90% or above in other provinces.^29^ By the end of 2015, Nyanza achieved near-saturation among males ages 15 to 49, attaining coverage of about 84%.^5^

The first 5 years comprised the catch-up phase, in which the program circumcised nearly 800,000 men. Today the country is in the process of rolling out its second VMMC strategy, to run from 2014 to 2019, via an approach that focuses on^30^:

- Maintaining momentum on the catch-up phase to reach all adult men who need VMMC
- Beginning phased roll-out of EIMC as a component of maternal, neonatal, and child health services
- Increasing VMMC coverage to at least 80% in all regions and to 95% nationally
- Lowering the adolescent target age to 10 years where demand is highest (from the current 14 years)

Although VMMC operated mainly as a parallel program during its first phase, the second-phase strategy is to integrate VMMC into the essential health package, ensuring local and sustainable financing.

## CONCLUSIONS

Regardless of the VMMC sustainability option that a country selects—focusing on circumcision of adolescents, EIMC, or a combination of both—financing availability, service delivery modalities, and systems-level support will be important to ensure that high male circumcision prevalence can be maintained beyond the initial coverage targets. Countries need to look ahead to the availability of human resources, commodities, supplies, demand creation, data availability, and more. Transitions in other health programs have taught us that early planning is critical. Countries can no longer put off the sustainability discussion until tomorrow. The time for that discussion is now.

Countries can no longer put off the sustainability discussion until tomorrow. The time for that discussion is now.

## Notes

**Open Peer Review**
